# Biocompatibility and patency of a novel titanium vascular anastomotic device in a pig jugular vein

**DOI:** 10.1038/s41598-021-97157-y

**Published:** 2021-09-01

**Authors:** Sanghyun An, Junsik Kim, Donghyun Lee, Minwoo Kim, Kangil Byun, Jangkyu Yun, Woonhyeok Jeong, Daegu Son

**Affiliations:** 1 Laboratory Animal Center, Daegu-Gyeongbuk Medical Innovation Foundation (DGMIF), Daegu, Republic of Korea; 2 Dentis Co. Ltd, Daegu, Republic of Korea; 3 Department of Plastic and Reconstructive Surgery, Keimyung University School of Medicine, Daegu, Republic of Korea

**Keywords:** Diseases, Medical research, Materials science

## Abstract

This study aimed to evaluate the biocompatibility and patency of our newly developed titanium vascular anastomotic device (TVAD) in a pig jugular vein. TVAD was made of commercially pure grade 2 titanium. The patency and anastomotic time were simultaneously confirmed in an ex-vivo system developed by the authors and in vivo using pig jugular veins. Five 8-month-old pigs, with body weights of 50–60 kg, underwent anastomosis of both jugular veins using the device. Graft patency was evaluated for 12 weeks by biplane angiography and sonography. All tissue biopsy samples were analysed by histology. In all 10 cases, the anastomosis was completed in < 5 min. The vessel lumen was not damaged, and the inner vessel wall was completely endothelialised at the anastomotic site. No foreign body reactions were observed at the vessel lumen, vessels, and outer vessel walls by histopathologic analysis. Patency and absence of leakage at the anastomotic site of the follow-up period were confirmed clearly by angiography and sonography. This preliminary animal study proved that our newly developed device is a very promising tool for intima-to-intima contact anastomosis. TVAD can be used as a feasible and safe medical tool for vessel anastomosis.

## Introduction

Traditional hand-suturing techniques require long operative time, high technical expertise, and use of complex instruments^1^. The reported anastomosis failure rate of 2–6% due to suturing errors may cause the failure of the whole reconstructive surgery and increase healthcare costs^2,3^. Therefore, alternative anastomotic techniques are highly needed to improve the precision and efficiency of the anastomosis process.

The last step in surgery is vascular anastomosis regardless of the types of procedures, including free flap or organ transplant. There could be anastomotic failures such as tearing, leaking, and narrowing of the lumen, through-stitching, and inclusion of the adventitia, although they decrease with improvement in the surgeon’s skill due to the learning curve associated with anastomosis^4,5^.

Regarding anastomosis, veins are easy to fail due to their thin and fragile vascular wall. Various types of vascular couplers for anastomosis without the use of sutures to reduce tissue ischaemic-reperfusion injury and organ warm ischaemic time as well as improve vascular patency and prevent life-threatening complications have been devised to overcome this failure^6–9^.

Vascular anastomotic devices have not been developed for organ transplantation yet. However, they have already been developed for microvascular applications for a vascular size of 1 to 4 mm^1,10,11^. These devices are mostly made of biocompatible absorbent plastic materials^12–14^, thus triggering concerns about the strength of the anastomosis in organ transplants^15–17^.

Titanium has become popular as a structural material for many medical devices due to its strong durability, light weight, and corrosion resistance^18,19^. These features are beneficial when transplanting an organ that needs the stability of vascular anastomosis and can withstand the weight of large vessels with stiffness.

Thus, we developed a large vascular anastomotic device using titanium. Then, we investigated the durability, anastomosis time, stability, and patency of our newly developed titanium vascular anastomotic device (TVAD) in a pig jugular vein for intravenous use.

## Materials and methods

### Design and fabrication of TVAD

TVAD was made of commercially pure grade 2 titanium because it is a good candidate for medical applications as it is strong and ductile enough to protect the blood vessels from tearing and results in less tissue reaction and fibrosis. TVAD is 0.21-inch thick and is designed to reduce the ischemic time and difficulty in performing the procedure, which may lead to a fast and less invasive vascular anastomosis*.*

The two important factors in designing a vascular anastomotic device are: (1) the part that secures each blood vessel to the device and (2) the two devices are combined. The first manipulation is called ‘hooking,’ which is similar to a fish hanging on a hook. The important aspect in hooking is that the blood vessels are attached to the device and maintained well until the operation is completed. The second manipulation is called ‘coupling.’ At this time, the blood should not leak into the gap between the connected devices.

**Figure 1:**
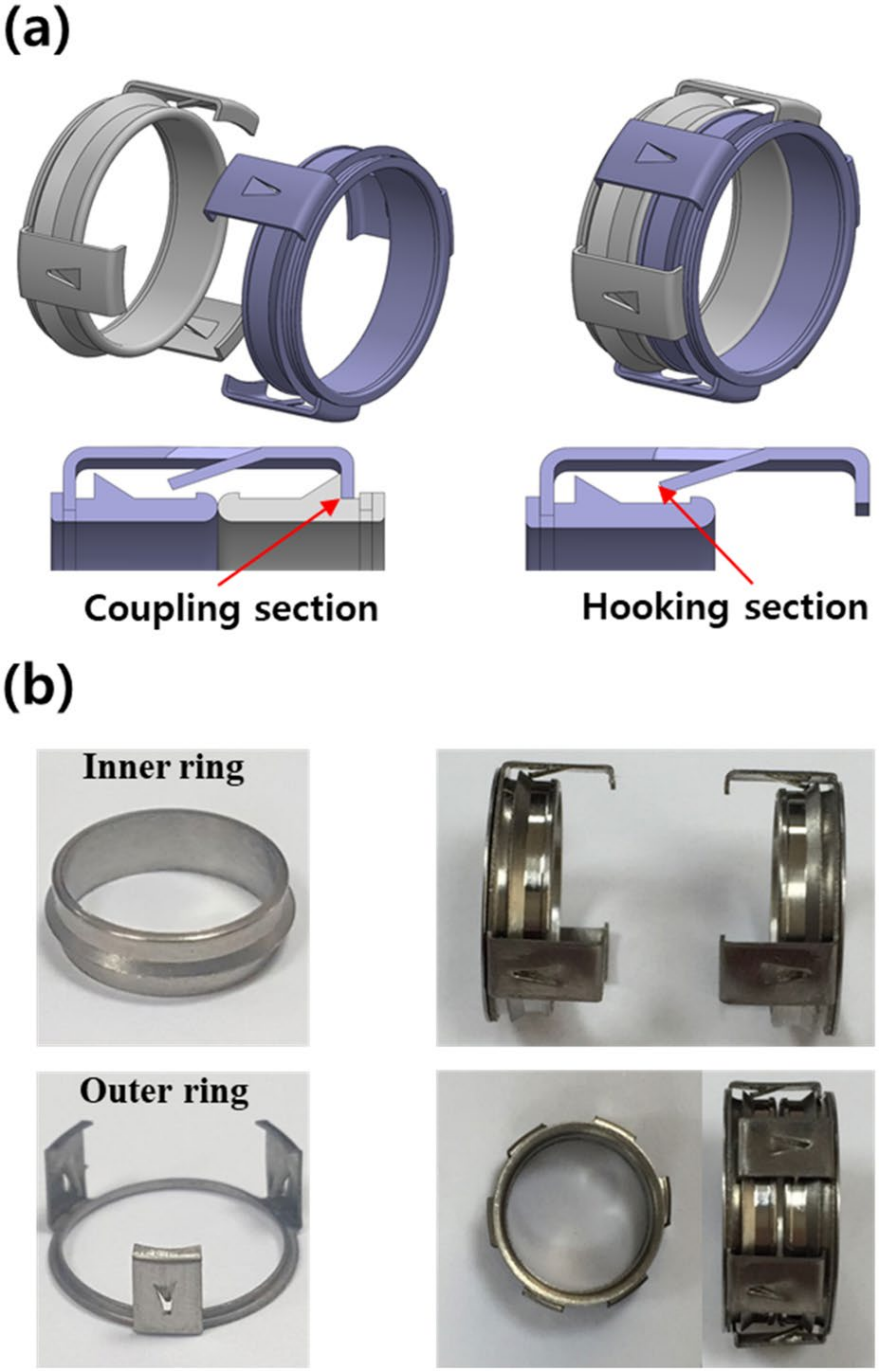
(**a**) Design of the titanium vascular anastomotic device (TVAD). TVAD comprises a hooking part that secures each blood vessel to the device and a coupling part that combines the two parts with a blood vessel. (**b**) Photographs of the actual parts assembled. The device comprises inner and outer rings. The inner ring has a protrusion on the body that connects with the connecting arm of the mating outer ring, and the outer ring comprises three connecting arms, with each arm having a triangular ring that hangs on the blood vessel.

The TVAD comprises inner and outer rings. The inner ring is made by a computer numerical control (CNC) machine, whereas the outer ring is made by a press machine. The inner ring has inclined projections on the body that connect with the connecting arm of the opposite outer ring, and both edges are rounded to prevent damage when blood vessels are joined. The outer ring comprises three connecting arms, and each arm has a triangular hook on the base that hooks the blood vessel. The triangular hook has a pointed end to penetrate the wall of the blood vessel and narrow the gap with the opposite inner ring to prevent the blood vessel from falling out during the operation. By combining these two rings, the final TVAD is created. In the case of coupling, if one TVAD is rotated 60°, the connection occurs at six locations at 60-degree intervals (Fig. 1). When the TVAD is initially connected to the vessel, the vessel is not rotated because the TVAD is already connected with the vessel in a 60-degree rotation state. Once the TVAD is combined, it can be removed using a specially designed instrument to ensure that the blood vessels are not damaged. Once removed, the TVAD cannot be reused again.

### Ex-vivo vascular anastomosis using TVAD

The qualitative assessment of leakage and the likelihood of tearing of the anastomosis with a coupler ex vivo testing was done using liquid saline.

The method of connecting the blood vessels with TVAD is as follows. First, the TVAD is mounted on the coupling loader, one vessel is inserted into the TVAD ring, and the vessel is turned over and inserted into three hooks spaced at 120°. The same operation is performed on the opposite blood vessel, and the loaders on both sides are in close contact with each other, allowing the connecting arms of the TVAD to push each other at 60-degree intervals to complete bonding. After the blood vessels are completely connected, the TVAD is separated from the coupling loader. The function of the coupling loader is to hold the rings during anastomosis. When the vascular anastomosis is completed, the TVAD is only on the outside of the blood vessel wall, not inside the blood vessel, and intima-to-intima contact is made between the ends of the blood vessel.

**Figure 2:**
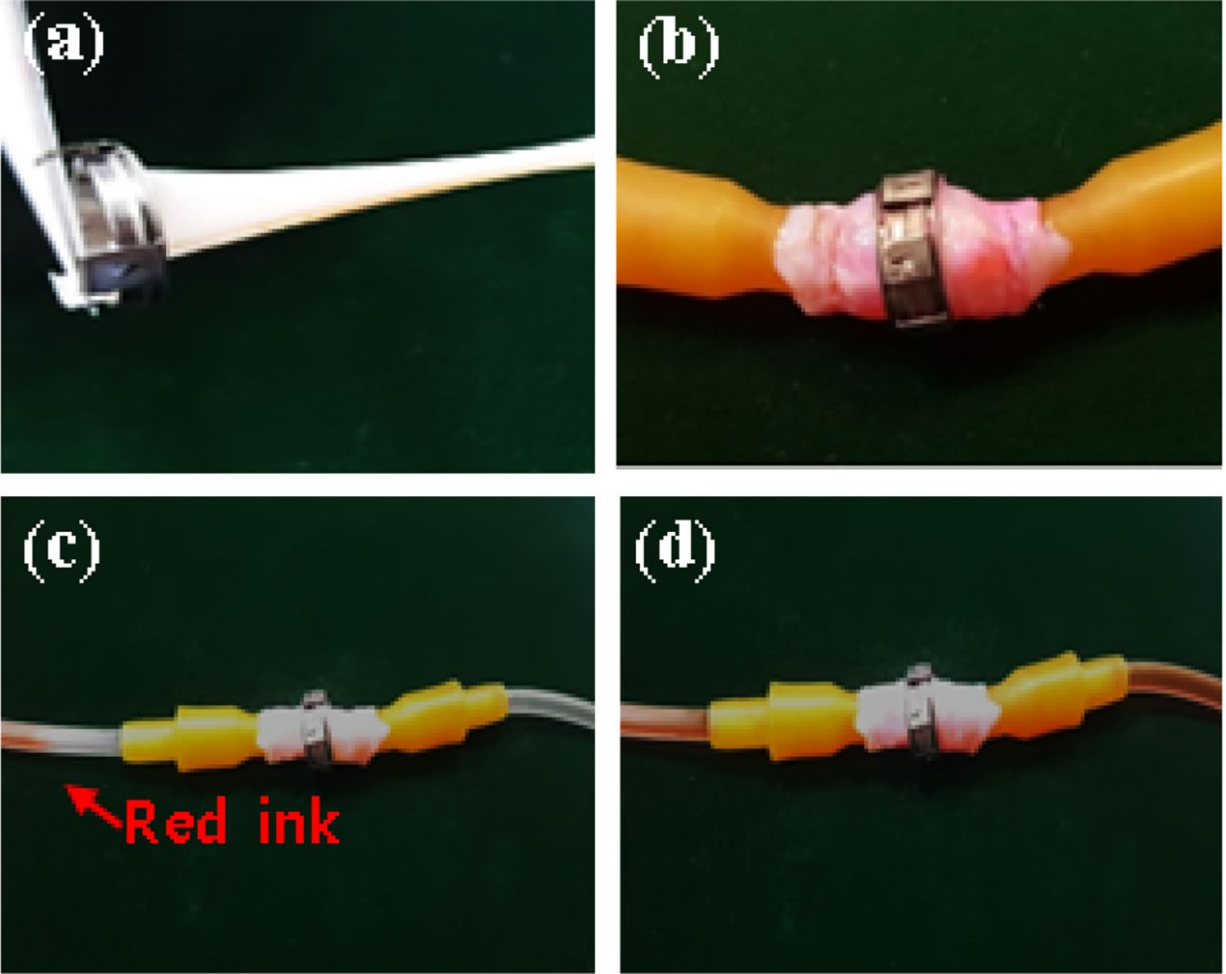
The qualitative assessment of leakage and the likelihood of tearing of the anastomosis with a coupler ex vivo testing was done using liquid saline. (**a**) The titanium vascular anastomotic device (TVAD) is mounted on the coupling loader; one vessel is inserted into the TVAD ring, and the vessel is turned over and inserted into three hooks spaced at 120°. (**b**) After the blood vessels are completely connected. (**c**) A normal saline bag mixed with red ink. (**d**) The pressure was adjusted according to the difference in height between the normal saline bag and the outlet.

The veins were harvested from cadaver pigs and tested when still fresh. A qualitative leakage test was performed on two pieces of porcine vessels connected with a 10-mm diameter TVAD. We performed the test using a normal saline bag mixed with red ink. One side of the intravenous set was connected to the saline bag, and one side was connected to the first jugular vein. After connecting the second jugular vein from another set, the two veins were anastomosed with the TVAD. The pressure was adjusted by the difference in height between the normal saline bag and the outlet (Fig. 2).

### In vivo vascular anastomosis using TVAD

**Figure 3:**
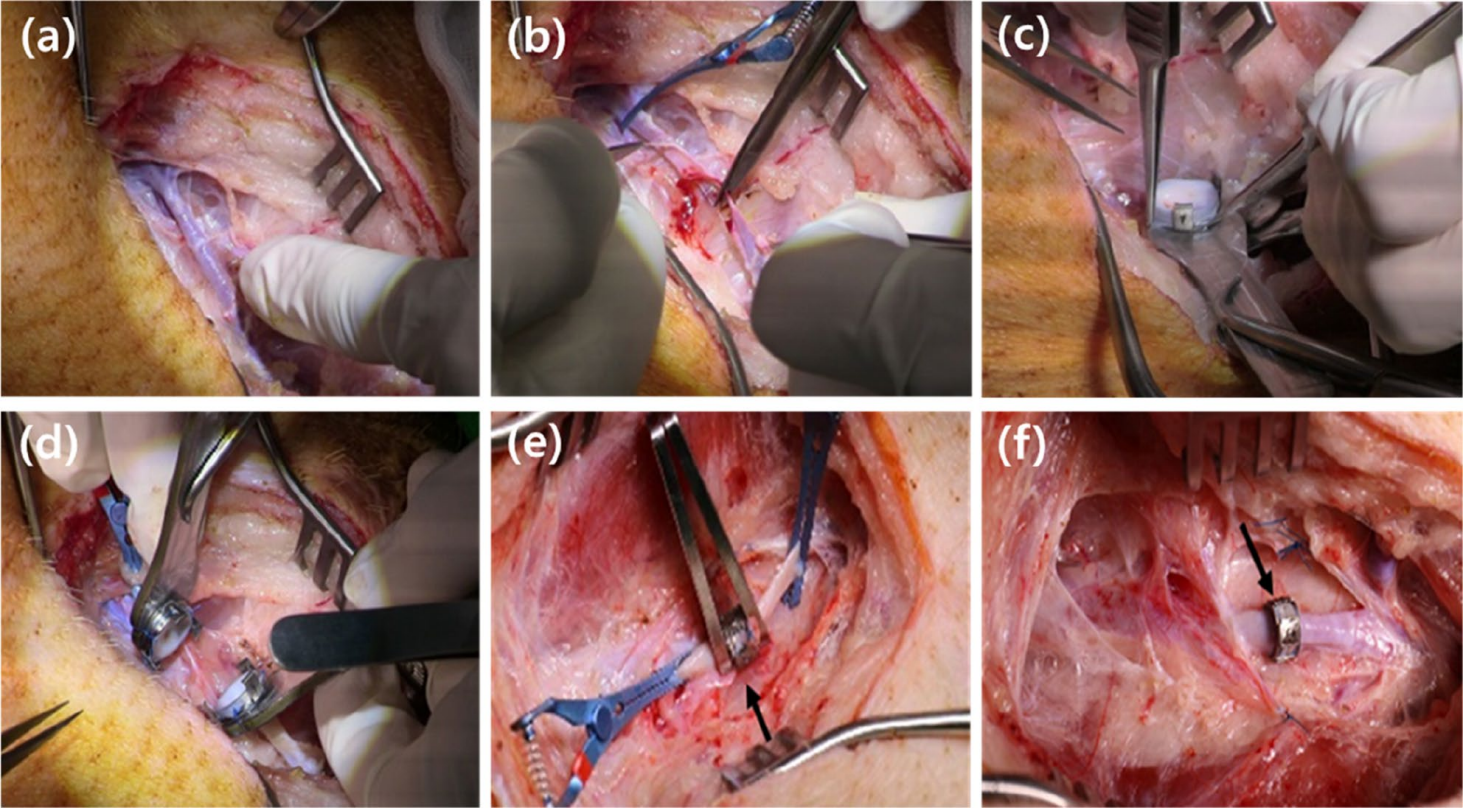
Sequential intraoperative photographs of the jugular vein anastomosis using titanium vascular anastomotic device (TVAD). (**a**) A longitudinal neck incision was made, and the external jugular veins in the neck were exposed. (**b**) After clamping both sides of the most cephalic and caudal areas of the exposed jugular vein, the blood vessel was vertically cut at the midpoint and divided. (**c**) One vessel is inserted into the TVAD ring, and the vessel is turned over and inserted into three hooks. (**d**) The same procedure is performed on the opposite blood vessel. The loaders on both sides are in close contact with each other. (**e**) The connecting arms of the TVAD push each other to complete bonding. (**f**) After complete coupling, the anastomosis is created.

To evaluate the functionality of the TVAD, end-to-end anastomosis was performed in pig external jugular veins. Five 8-month-old pigs, with body weights of 50–60 kg, underwent anastomosis of both jugular veins using the TVAD with a coupling loader. All animal procedures were performed using the protocols approved by the Institutional Animal Care and Use Committee of Daegu-Gyeongbuk Medical Innovation Foundation (approval number: DGMIF-16030804-00). Besides, all procedures were conducted in compliance with the ARRIVE (Animal research: reporting of in vivo experiments) guidelines (http://www.nc3rs.org.uk/page.asp?id=1357), and all efforts were made to minimize the number of animals and induced pain. The five pigs were anaesthetised by an intramuscular injection of 5 mg/kg of zolazepam (Zoletil 50, Virbac Korea, Seoul, Korea) and 2 mg/kg of xylazine hydrochloride (Rompun, Bayer, Leverkusen, Germany) after fasting for 12 h and abstinence from water for 6 h. After intubation, anaesthesia was maintained with 1.2–2.04% minimum alveolar concentration (MAC) of isoflurane during the surgery. With the pigs placed in a supine position, the upper abdomen and neck area were shaved and draped with sterile towels. Normal saline (2–4 mL/kg/hour) was infused through the venous cannula in the auricular vein during surgery to maintain adequate preload stability. Through a longitudinal incision on the neck, the jugular veins were exposed. The jugular veins were dissected from the perivascular tissue. After clamping on both sides of the most cephalic and caudal areas of the exposed jugular vein, the blood vessel was transversally cut at the midpoint and divided. The blood clot in the blood vessel was washed, and adventectomy was performed at both ends of the vessel. Vascular anastomosis was performed as described above, and a 10-mm diameter TVAD was used (Fig. 3).

Given that both ends of the cut blood vessel are turned over and fixed on the TVAD, they are slightly shorter than the length of normal blood vessel. In addition, the length of the blood vessels required for the eversion is approximately 2 mm on each end of the cut. Due to these situations, tension occurs in the anastomosis, and to prevent this, the blood vessel walls are pulled and sutured together at the proximal and distal areas of the anastomosis to relieve tension.

### Examination of patency and leakage in anastomotic vessels

The ultrasonographic and angiographic assessment of vascular patency was performed on an ultrasound system (Sonoace7; Samsung, Korea) at 1, 4, 8, and 12 weeks after anastomosis. All animals were anaesthetised by intramuscular administration of a mixture of zolazepam and xylazine for immobilisation. A lubricant was applied to the surgical area, and duplex ultrasonography was performed in the sagittal and axial planes to evaluate the vessel’s patency and the presence of vessel leakage or clot formation. At 12 weeks, vascular patency was evaluated by a biplane high-resolution angiography system (Axiom Artis zee; Siemens, Germany). The observation period of 12 weeks was determined because it was long enough to confirm the healing of the tunica media of the anastomotic site. A 5-Fr intravascular sheath was inserted into the right femoral vein in pigs. Angiography was performed using a 5-Fr JB1 intra-arterial catheter (Cook Canada, Stouffville, ON, Canada) at the anastomotic jugular vein. Iopamidol (Iopamiro 300, Bracco, Italy) was used for vessel contrast. After angiography, all pigs underwent open exploration to determine the patency of the anastomoses, followed by a biopsy to obtain specimens.

## Results

### Ex vivo analysis of vascular anastomosis using TVAD

The TVAD is designed to increase reliability and reduce the amount of time spent connecting two vessels. A qualitative assessment of leakage and likelihood of tearing of the anastomosis was performed through the device connected to the porcine veins in an ex vivo bench testing. Figure 2 shows the final status of the process. The mean duration of ex vivo anastomosis construction using the loaders was 176 ± 58 s. No leakage and flow resistance occurred at the coupling point of the vessel during the whole process on internal pressures of up to 51.71 (one psi) mmHg.

### In vivo analysis of vascular anastomosis using TVAD

To evaluate the functionality, the TVAD was inserted easily into both jugular veins of the five pigs using a coupling loader with a 10-mm inner diameter. The experiments were successful on all five pigs. The TVAD was used, and no technical failure was detected. In all 10 anastomoses, the feasibility and reproducibility of this new system were confirmed. The new device enhances the ease of performing the procedure and reduces the risk of tearing the vessel wall and thrombosis by passing the vessel into the ring, thereby everting the edges and ensuring intima-to-intima contact. When the vascular clamps were released, blood leakage was not observed in the vein in the application sites, as shown in Fig. 3 and Supplementary Videos 1. In Table 1, the times for completion of the anastomosis are presented. The average anastomosis time was 267 ± 35 s. No intra-operative or post-operative complications and evidence of immediate thrombosis, occlusion, or embolus were observed in the application pigs.

**Table 1:** Anastomotic time using a titanium vascular anastomotic device (TVAD).

Animal Number	Anastomosis Time (Sec)	Mean	SD
SP1	199	267.80	35.05
SP2	251		
SP3	278		
SP4	247		
SP5	276		
SP6	341		
SP7	271		
SP8	276		
SP9	269		
SP10	270		

### Anastomotic patency and leakage examination using imaging

**Figure 4:**
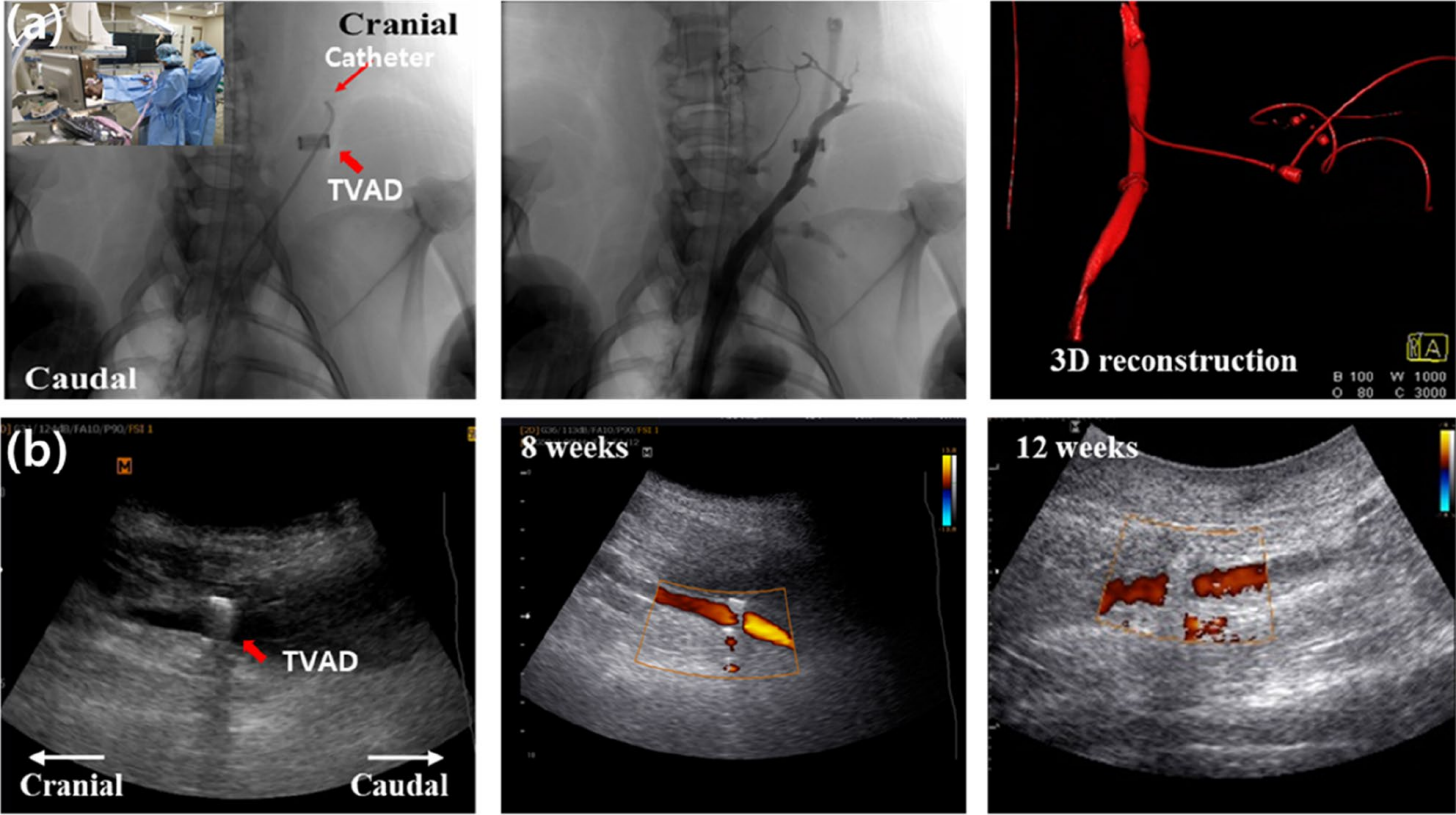
Representative angiographic and ultrasonographic images at 12 weeks after the anastomosis using titanium vascular anastomotic device (TVAD). (**a**) Conventional angiography demonstrated excellent patency of the anastomosis without stenosis caused by ischaemic change from the injured vasa recta. (**b**) Ultrasonographic examination also revealed a patent flow at the anastomotic site before and after the procedure, although it is limited to evaluating the flow at the anastomosis site that is hidden by posterior shadowing of the titanium coupler.

At 12 weeks after the procedure, the weight of each TVAD pig was similar to the average weight of normal pigs. There was no weight loss due to physical stress, such as infection. All pigs successfully recovered after anastomosis. Patency results at the anastomotic site were good by angiography and ultrasonography at 12 weeks post-operatively. Figure 4 shows no angiographic (Fig. 4a) and ultrasonographic (Fig. 4b) signs of narrowing, including leakage from the anastomosis, immediate haemostasis, adequate blood flow, and instances of technical failure at the anastomotic site.

Angiography with 3D reconstruction using a contrast material is crucial to confirm any leakage at the anastomotic site. There was no leakage of the contrast material through the access tract into the neck. Ultrasonographic examinations at 1, 4, 8, and 12 weeks after surgery confirmed adequate blood flow at the proximal and distal sites of TVAD.

### Histological and macro-photographic examinations

**Figure 5:**
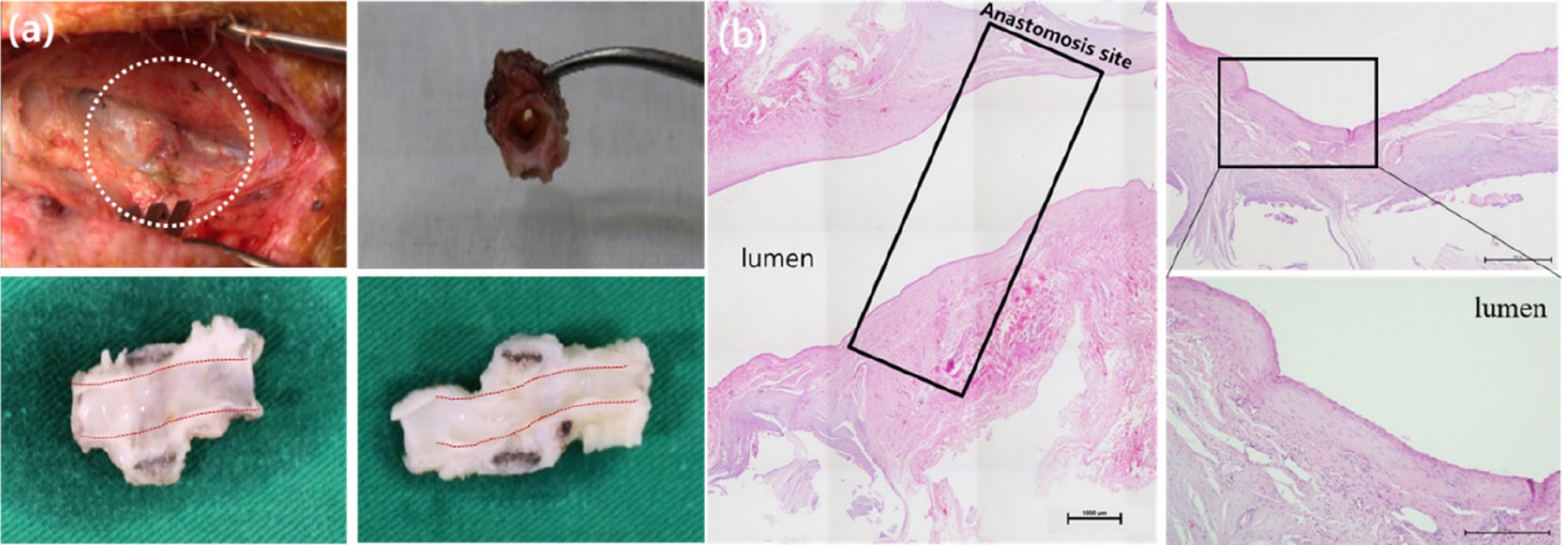
Representative (**a**) macro-photographic image and (**b**) haematoxylin and eosin staining at 12 weeks postoperatively. Macro-photographic and histological and findings of the vascular connection site showed that the titanium vascular anastomotic device (TVAD) was well attached to the outer wall of the vessel, the vessel lumen had no damage, and the inner wall of the vessel was completely endothelialised. There was no evidence of a profound foreign body reaction at the site of anastomosis when the TVAD was used. Moreover, the intima-to-intima contacts were clear (the region containing the anastomotic site is indicated by a rectangle).

All pigs were sacrificed at 12 weeks after the surgery. Histological and macro-photographic findings of the vascular connection site showed that TVAD was well attached to the outer wall of the vessel, the vessel lumen had no damage, and the inner wall of the vessel was completely endothelialised. There were no foreign body reactions in the vessel lumen, vessels, and outer walls of the vessels, TVAD was completely enclosed in capsules, and no inflammation was observed anywhere, as shown in Fig. 5. Therefore, the inner diameter of the blood vessels did not show narrowing at the vascular connection site, and smooth regeneration was achieved. The anastomosis site was clean, without any debris.

## Discussion

Vascular anastomoses are used by plastic surgeons not only for reconstructive microsurgery but also for other procedures, including organ transplantation as well as cardiac, vascular, and neurosurgeries^20–22^. Carrel established four main guidelines for vascular anastomoses in 1902^23^. The guidelines recommended avoiding luminal narrowing at the anastomotic site, avoiding the creation of folds and a rough inner surface of the vessel, and affixing the two intimal edges closely. Moreover, it was suggested that it is preferable to eliminate contact of suture material with blood. This principle is equally important today. However, most suture materials are in direct contact with blood in the blood vessels^24,25^.

After introducing the microvascular anastomotic coupling device (MACD) in the 1980s, the widely cited advantages of the MACD include intima-to-intima anastomosis and reliable vessel eversion, thereby avoiding a potentially thrombogenic foreign body within the vessel lumen^10,26^. Therefore, we developed a new simple anastomotic device capable of uniting vessel diameters to ensure intima-to-intima contact.

Blood vessels must always be resilient to adequately supply blood to the organs. In general, non-absorbable suture materials have been used to connect blood vessels, given that absorbable sutures have been considered incapable of providing sufficient resistance to tension. Therefore, to improve the mechanical property and reduce the foreign-body reaction, a non-absorbable metallic anastomotic device is required. However, the anastomotic site will always maintain a constant vascular diameter due to the metal device. Therefore, if the priority is organ blood supply, an absorbable anastomotic device would be suitable because when the absorbable anastomotic device is completely resorbed after a complete normal vessel remodeling, the anastomotic site regains elasticity. However, if the absorbable anastomotic devices cause severe inflammation and foreign body reaction in vivo before a sufficient remodeling of the blood vessels, leakage may occur at the anastomotic site^27–29^. This risk must be completely overcome to allow the successful use of absorbable anastomotic devices.

For the metallic device used in the procedures, a nickel-titanium alloy is commonly used. However, the development of a nickel-free titanium-based device for biomedical applications is still ongoing because nickel is a toxic element. Generally, the nickel composite has been known to cause cell toxicity and modifications in cellular level activity. When a nickel composite device is implanted in the body, nickel ions are released to the surrounding tissue from the device and act as cofactors or inhibitors in enzymatic processes, obstruct intracellular organelles, transform cell morphology, and reduce cell numbers^30^. Moreover, recent studies have reported that patients who received nickel-containing implant devices had tissue inflammation^31^. Therefore, this paper demonstrates the feasibility of performing suture-less end-to-end anastomoses using a pure titanium anastomotic device.

The most important features of our device are its “hooking” and “coupling” parts. The preliminary ex vivo test greatly contributed to the improvement of our device, which was completed in a short period, because the test allowed us to identify problems in the two crucial features and fix these issues immediately; then, the device was applied again. In the design of the non-absorbable vascular anastomotic device, special consideration is required for the area where the device surrounds the vascular wall. This is because the blood supply from the outside is blocked at the site. Looking closely at our device, a small hole is drilled around the TVAD, which induces vascular ingrowth from the outside. Our device was developed specifically to connect veins and is not suitable for arteries because the thinner the blood vessel, the more suitable it is to fix the vessel by turning it over.

To evaluate the functionality of the TVAD, end-to-end anastomosis was performed on a pig model. Leakage or stenosis was not detected during the observation period in the animal models. Early anastomotic leakage of the vessels after the operation is considered to have many potential causes. In particular, if the blood vessel is too compressed by the metal material, there is a risk of damage, and if it is too loose, leakage occurs; thus, it is imperative to determine the appropriate gap or tolerance to apply during the procedure. In a clinical situation, potential leakage or tearing by unmated devices could be identified immediately after completing the anastomosis. In this case, profuse bleeding occurs at the anastomotic site. However, delayed separation of anastomotic coupler could arise from excessive movement and tension at the anastomosis site. This problem is a universal challenge for anastomotic devices. Although we used a small number of experimental models in this study, we did not experience disruption of anastomotic devices immediately after the operation and throughout the follow-up period.

In the angiographic and ultrasonographic assessment scanning at 12 weeks postoperatively, anastomotic sites were clearly patent without stenosis and leakage in pigs. Histologically, it was confirmed that the endothelialization was complete at the intima-to-intima contact site. The regenerated intima around the anastomosis site was smooth with no fibrin deposition. It seemed that our device did not interfere with the healing of the anastomosis site and endothelialization.

The observation period of 12 weeks may seem inadequate. In other experiments performed by the authors^26^, we observed healing of the tunica media of the anastomotic site at 12 weeks and maturation at 22 ~ 26 weeks. Therefore, we have selected 12 weeks for the healing of the tunica media. According to the authors' previous research experience, if there are no problems until 12 weeks, better results through maturation are expected.

The anastomotic time of our device was < 5 min. Although there is no comparative study, considering that it usually takes 20–30 min to connect a single blood vessel, our blood vessel anastomotic time was significantly shortened^32^. Increased ischaemia and operative times are known to increase the risk of complications in microsurgery, particularly in older patients^33^. In particular, in organ transplantation, an increase in warm ischaemic time has an absolute effect on the success or failure of surgery^34^.

Locally, increased ischaemic time leads to tissue injury and may progress to ischaemia–reperfusion injury or compartment syndrome. Systemically, prolonged operative time has been shown to significantly increase the complication rate. An improvement that can safely decrease ischaemia or operative time while maintaining an effective and biocompatible vessel anastomosis would positively benefit patients.

## Conclusions

Through this preliminary animal study, we proved that our newly developed device is a promising tool for intima-to-intima contact anastomosis. The TVAD can be used as a feasible and safe medical tool for vessel anastomosis in the field of vascular surgery and organ transplantation. To support the evidence for the advantages of applying TVAD in vascular anastomosis, further research and clinical investigations should be conducted.

## Supplementary Information

Supplementary Video 1.

Supplementary Video 2.

Supplementary Information 1.

## Data Availability

All data generated or analysed during this study are included in this published article and its Supplementary Information files.
