# Supplementary material for: Biocompatibility and patency of a novel titanium vascular anastomotic device in a pig jugular vein
Source: Sci Rep. 2021 Sep 1;11:17512. doi: 10.1038/s41598-021-97157-y (PMC8410932; doi:10.1038/s41598-021-97157-y)
Supplement: Supplementary file 3 — Supplementary Information 1. [file 41598_2021_97157_MOESM3_ESM.pdf]

Biocompatibility and Patency of a Novel Titanium Vascular Anastomotic Device in a Pig Jugular Vein

Sanghyun An[1], Junsik Kim[1], Donghyun Lee[1], Minwoo Kim[2], Kangil Byun[2], Jangkyu Yoon[2], Woonhyeok Jeong[3], Daegu Son[3,*]

1 Laboratory Animal Center, Daegu-Gyeongbuk Medical Innovation Foundation (DGMIF), Daegu, 41061, Republic of Korea

2 Dentis Co. Ltd, Daegu, Republic of Korea

3 Department of Plastic and Reconstructive Surgery, Keimyung University School of Medicine, Daegu, Republic of Korea

* Correspondence: Daegu Son, M.D., Ph.D., Tel.: +82 53 258 7817; Fax: +82 53 258 4590

E-mail address: handson@dsmc.or.kr

Supplementary Video 1.

No blood leakage was observed in the jugular vein using the titanium vascular anastomotic device when the vascular clamp was released.

Supplementary Video 2.

Easily and perfectly coupled with a clicking sound at the titanium vascular anastomotic device coupling.
